# Piperlongumine and immune cytokine TRAIL synergize to promote tumor death

**DOI:** 10.1038/srep09987

**Published:** 2015-05-18

**Authors:** Jiahe Li, Charles C. Sharkey, Michael R. King

**Affiliations:** 1 Department of Biomedical Engineering, Cornell University, Ithaca, New York, USA, 14853

## Abstract

Malignant transformation results in increased levels of reactive oxygen species (ROS). Adaption to this toxic stress allows cancer cells to proliferate. Recently, piperlongumine (PL), a natural alkaloid, was identified to exhibit novel anticancer effects by targeting ROS signaling. PL induces apoptosis specifically in cancer cells by downregulating several anti-apoptotic proteins. Notably, the same anti-apoptotic proteins were previously found to reduce tumor necrosis factor-related apoptosis-inducing ligand (TRAIL)-induced apoptosis in cancer cells. Therefore, we reasoned that PL would synergize with TRAIL to stimulate potent apoptosis in cancer cells. We demonstrate for the first time that PL and TRAIL exhibit a synergistic anti-cancer effect in cancer cell lines of various origins. PL resulted in the upregulation of TRAIL receptor DR5, which potentiated TRAIL-induced apoptosis in cancer cells. Furthermore, such upregulation was found to be dependent on ROS and the activation of JNK and p38 kinases. Treatment with combined PL and TRAIL demonstrated significant anti-proliferative effects in a triple-negative breast cancer MDA-MB-231 xenograft model. This work provides a novel therapeutic approach for inducing cancer cell death. Combination of PL and TRAIL may suggest a novel paradigm for treatment of primary and metastatic tumors.

Most FDA-approved cancer chemotherapy agents are broadly categorized into “proliferation inhibitory” and “signaling targeted” drugs. Proliferation inhibitory drugs gain their selectivity by targeting rapidly dividing cancer cells. These drugs have been found to inhibit mitosis or prevent DNA replication. For example, paclitaxel and related drugs that inhibit microtubule polymerization dynamics have proven effective for treating some epithelial cancers (breast, lung, prostate and others)^1^. In contrast, nucleoside analogues or DNA-intercalating agents, such as doxorubicin, target proliferating cells by interfering with the synthesis of genomic DNA^2^. Nevertheless, normal tissues or organs such as bone marrow, gut and hair follicles undergo rapid division making them susceptible to the anti-proliferative effect of these drugs^3^,^4^,^5^. Such toxicities can prevent continuous dosing of the drugs in certain patients with relatively high sensitivity to this side effect.

A second class of drugs inhibits specific signaling pathways that can promote tumor growth^6^. These signaling pathways are commonly amplified in certain cancers through protein overexpression or gene amplification, while these pathways in normal cells are not^7^,^8^. One example of this class is tyrosine kinase inhibitor. Trastuzumab, a humanized anti-HER-2 monoclonal antibody, has been approved for treatment of patients with breast cancers that overexpress the human epidermal growth factor receptor-2 (HER-2) protein or that exhibit ErbB2 gene amplification^9^. However, the fact that the overexpression of HER-2 occurs in 25-30% of breast cancers renders the therapy ineffective against the remainder of breast cancer cells that do not rely on HER-2 overexpression^10^.

Recently, a new class of chemicals targeting the stress response to reactive oxygen species (ROS) has gained attention due to the chemicals’ specificity and broad effect in a variety of cancer types^11^. It is postulated that malignant transformation, caused by gain-of-function activation in oncogenes or loss-of-function mutations in tumor suppressor genes, results in enhanced cellular stress. Adaptation to this stress is necessary for cancer cell survival while it is not required in normal cells^12^. Using a high throughput small-molecule screening, piperlongumine (PL) was identified as a drug that increases the level of ROS and apoptosis in cancer cells but has little inhibitory effect on either rapidly or slow dividing normal cells^13^. It was later shown that PL also induces autophagy in cancer cells as an alternative strategy of suppression in caspase-independent cancer cell death^14^. A biochemical examination of PL-induced cell death indicated that it represses various anti-apoptotic proteins including B-cell CLL/lymphoma 2 (BCL2), baculoviral IAP repeat-containing 5 (also known as survivin) and X-linked inhibitor of apoptosis (XIAP) in cancer cells^13^.

Clinical treatments involving a single drug have proven short-lived due to the emergence of resistant cancer cells. A major strategy for overcoming drug resistance is combination therapy^15^,^16^,^17^. Given the effectiveness of PL in cancer cell lines and preclinical mouse models, it remains to be answered if a synergistic effect or enhanced induction of apoptosis can be achieved when PL is combined with another drug. One reagent explored in this study is tumor necrosis factor-related apoptosis-inducing ligand (TRAIL), which is under clinical trials for cancer therapy^18^. Similar to PL, TRAIL has been found to kill cancer cells while sparing normal cells^19^,^20^,^21^. It is broadly expressed on lymphocytes, neutrophils and other immune cells as an innate immune cytokine to clear diseased cells. TRAIL can induce apoptosis immediately following its binding to TRAIL receptors highly expressed in cancer cells of various origins^19^. However, certain cancer cells develop TRAIL resistance through elevated expression of anti-apoptotic proteins that are nevertheless suppressed by PL^13^,^22^. In this study, we investigated the effect of these two combined tumor-specific reagents on cancer cells and normal cells. Furthermore, the preclinical efficacy of the combination therapy was evaluated in a triple-negative breast cancer model.

## Results

### The synergistic anti-tumor effect of PL and TRAIL

**Figure 1:**
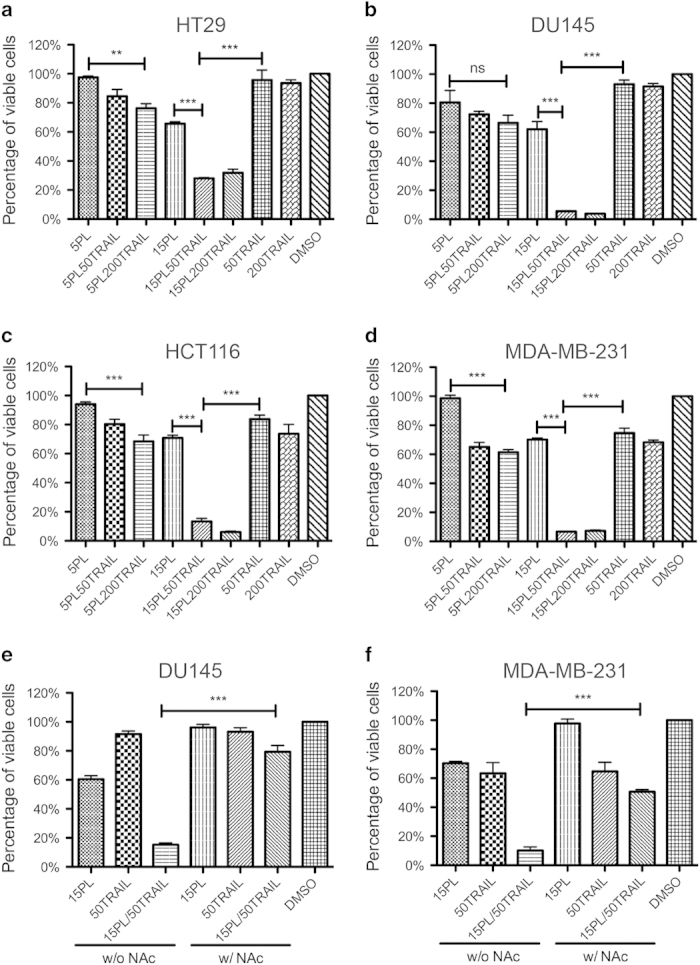
The anti-tumor effect of combined PL and TRAIL. Cancer cells were treated with the indicated concentrations of PL (5 or 15 μM) and/or TRAIL (50 or 200 ng/mL) in 48-well plates for 24 hr. Cell viability was measured using MTT assay. The cell lines tested were: **(a)** HT29 (colon cancer), **(b)** DU145 (prostate cancer), **(c)** HCT116 (colon cancer) and **(d)** MDA-MB-231 (breast cancer). **(e,f)** PL-induced ROS mediated synergistic anti-proliferation effect of PL and TRAIL. Pretreatment of DU145 and MDA-MB-231 with 1 mM NAc significantly increased viability of cells subjected to combined PL and TRAIL, with cell viability close to TRAIL treatment alone. All results are presented as the mean ±SEM, n = 3; **, p < 0.01; ***, p < 0.001; ns, no significant difference.

The effect of PL and TRAIL combination treatment on cell viability was tested in colon cancer (HT29 and HCT116), prostate cancer (DU145) and breast cancer (MDA-MB-231) cells. The concentrations of PL and TRAIL were chosen based on previous studies^23^,^24^. Following 24 hr of treatment with PL, TRAIL, or a combination of both, the percentage of viable cells significantly decreased compared to the single treatments in a dose-dependent manner measured by MTT assay (Fig. 1a–d) and crystal violet assay (Figure S1). Furthermore, PL and TRAIL exerted a synergistic inhibitory effect in these tested cell lines as evaluated by Jin’s formula (Figure S2). Such synergy was observed when 15 μM PL was combined with 50 or 200 ng/mL TRAIL. Given the role of PL in raising ROS level in transformed cells as previously reported^13^, it remained unclear whether PL potentiated TRAIL-induced growth inhibition via ROS. To generalize the mechanism to multiple cell types, we used one prostate cancer cell line (DU145) and one breast cancer cell line (MDA-MB-231). In addition, DU145 and MDA-MB-231 were chosen based on their differential sensitivity to TRAIL. DU145 and MDA-MB-231 cells were pretreated with 1 mM ROS scavenger N-acetyl Cysteine (NAc) followed by treatment with PL, TRAIL, or their combination. Whereas NAc abolished the cytotoxic effect of PL, it failed to affect TRAIL activity. Moreover, pretreatment with NAc completely negated the synergistic effect of combined PL and TRAIL, with cell viability close to TRAIL treatment alone (Fig. 1e,f).

We next determined the effect of PL on ROS levels in these two cancer cell lines through flow cytometry using the redox-sensitive fluorescent probe CM-H2DCFDA. Treatment with PL for 6  hr caused a marked increase in ROS levels in comparison to DMSO treatment. In contrast, pretreatment with 1 mM NAc fully reversed the PL-induced ROS elevation (Figure S3). Therefore, it is concluded that the observed synergy of combined PL and TRAIL on cancer cells is mediated by ROS.

Given that certain cancer drugs such as doxorubicin (Dox) also generate oxidant stress and may synergize with TRAIL via ROS, we further sought to determine whether the mechanisms of TRAIL sensitization in cancer cells by PL and Dox converge on ROS production. Dox was found to increase ROS in both DU145 and MDA-MB-231 after 6 hr of incubation with cancer cells. In contrast, pretreating cells with 1 mM NAc followed by Dox yielded reduced ROS levels equivalent to the basal level seen in cells not treated with Dox. As expected, we further demonstrated that Dox can synergize with TRAIL to inhibit cell proliferation in these two cancer cell lines to an extent close to the PL and TRAIL combination (Figure S4a). Such synergy was also found to be ROS-dependent, since pretreating cancer cells with 1 mM NAc abolished the synergistic effect of Dox and TRAIL (Figure S4b). However, despite the fact that NAc could neutralize the inhibitory effect of a single PL treatment on cell viability (Fig. 1e,f), a single Dox treatment was still effective in reducing cell viability in the presence of NAc (Figure S4b). Based on these data, one may conclude that both PL and Dox are dependent on ROS to synergize with TRAIL for enhanced apoptosis induction. Additionally, ROS drives the toxicity of PL by itself, whereas Dox does not rely on ROS for its own cytotoxicity.

### TRAIL enhances PL-induced apoptosis signaling

**Figure 2:**
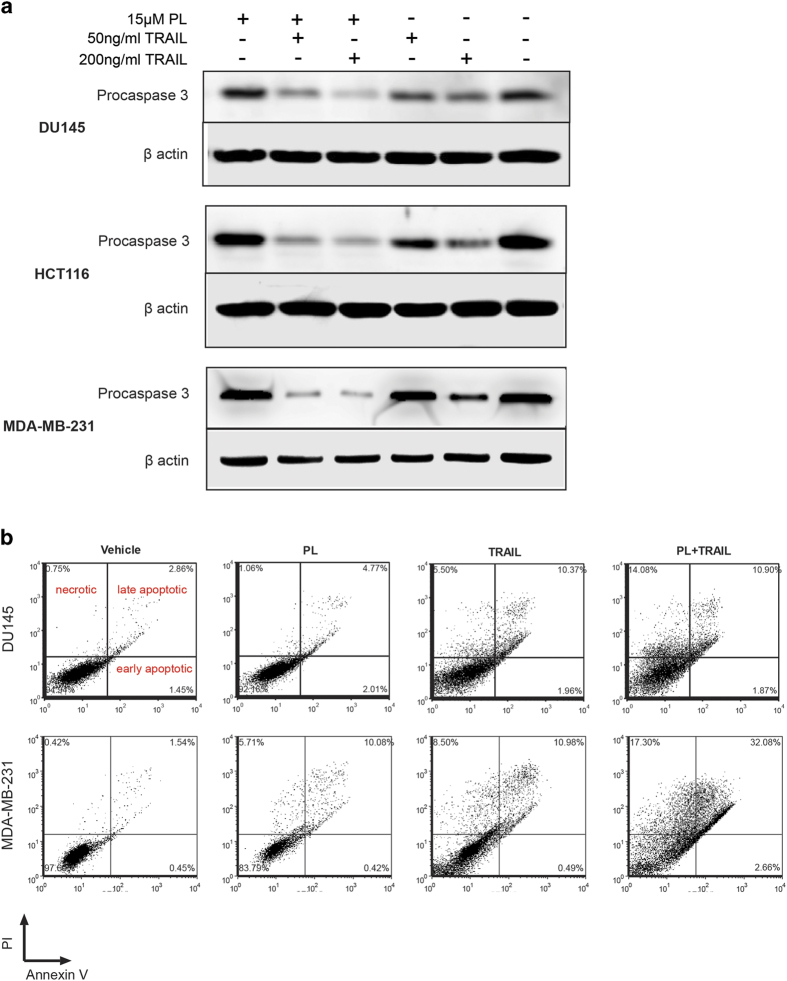
Enhancement of PL-induced apoptosis by TRAIL. **(a)** DU145, HCT116 and MDA-MB-231 were treated with the indicated concentrations of PL and TRAIL in 6-well plates for 12 hr. The cleavage of procaspase 3 was detected by anti-procaspase 3 in total lysate. Cropped images of western blotting are shown. **(b)** Flow cytometry of annexin V/PI staining of apoptosis/necrosis. DU145 and MDA-MB-231 cells were treated with DMSO, 15 μM PL, 50 ng/ml TRAIL or combined PL and TRAIL for 4 hr. Necrosis, early and late apoptosis events were detected by annexin V/PI. Representative dot plots from two experiments are shown.

Although previous studies showed that PL can induce apoptosis selectively in cancer cells, it has not been previously shown whether TRAIL can enhance PL-induced apoptosis. The cleavage of procaspase 3, which indicates the activation of apoptosis signaling, was examined by western blotting in the DU145, HCT116 and MDA-MB-231 cancer cell lines. In contrast to minimal cleavage of procaspase 3 by PL, TRAIL significantly enhanced cleavage of procaspase 3 when combined with PL (Fig. 2a). Furthermore, an annexin V/PI assay was performed to differentiate necrotic, late apoptotic and early apoptotic cells after treatments of DMSO, 15 μM PL, 50 ng/ml TRAIL or combined TRAIL and PL in DU145 and MDA-MB-231 cells for 4 hr. In DU145, it was shown that the combined cell deaths (necrosis, late apoptosis and early apoptosis) were induced at 2.7% by PL, 12.8% by TRAIL, and 21.8% via combined PL and TRAIL after subtracting basal cell death in the control treatment. For MDA-MB-231, the combined cell death percentages were 13.7% by PL, 17.6% by TRAIL, and 49.5% via the combination of PL and TRAIL (Fig. 2b).

### PL upregulates death receptor 5 (DR5) via ROS

**Figure 3:**
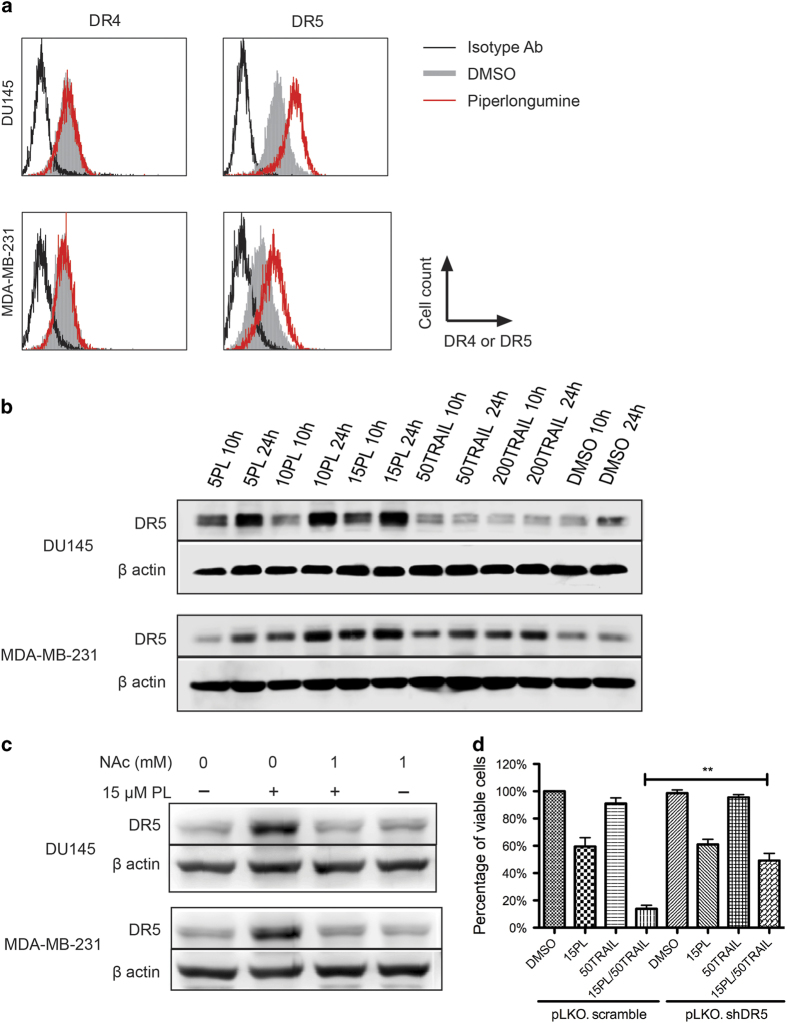
Piperlongumine upregulates expression of TRAIL receptor DR5. **(a)** Flow cytometry analysis of DR4 and DR5 expression in DU145 following treatment with DMSO or 15 μM PL for 6 hr. In contrast to DR4, DR5 expression on the cell surface was significantly elevated by PL. **(b)** Total DR5 expression induced by PL or TRAIL in a concentration- and time-dependent manner. DU145 and MDA-MB-231 were treated with PL (5, 10, 15 μM) or TRAIL (50 and 200 ng/mL) for 10 and 24 hr. Total DR5 protein was examined by western blotting. Whereas PL elevated DR5 expression upon increase of concentration and incubation time in both cell lines, TRAIL by itself only upregulated DR5 in MDA-MB-231. Cropped blots are shown. **(c)** Pretreatment of cancer cells with antioxidant NAc for 1 hr abolished the upregulation of DR5 by PL. The expression of total DR5 was measured by western blotting following incubation with 1 mM NAc and/or 15 μM PL. Cropped blots are shown. **(d)** Silencing DR5 expression reduced the synergistic anti-proliferation effect of combined PL and TRAIL. DU145 cells stably transduced with scrambled shRNA (pLKO. scramble) or DR5 shRNA (pLKO. shDR5) were treated with PL, TRAIL, and their combination at the indicated concentrations. Cell proliferation was measured by MTT assay. Results are mean ± SEM, n = 3; **, p < 0.01.

TRAIL-induced apoptosis signaling is triggered by the engagement of soluble TRAIL with TRAIL receptor DR4 and/or DR5 expressed on cancer cells. Previous studies showed that certain cancers develop TRAIL resistance by downregulating DRs^22^. In light of the enhanced anti-tumor effect of combined PL and TRAIL, it is not clear whether PL also modulates the expression of DRs in addition to the downregulation of anti-apoptotic proteins as reported previously^13^. Expression of DR4 and DR5 at the transcriptional level was first investigated. Little change of mRNA (less than 2-fold difference) was observed after cells were exposed to 15 μM PL over 6, 12 and 24 hr (Figure S5). The surface expression of DR4 and DR5 were examined by flow cytometry in DU145 and MDA-MB-231 cells following 10 hr of exposure to 15 μM PL. Whereas DR4 expression was unaffected, expression of DR5 was significantly elevated by PL compared to vehicle control (Fig. 3a). Furthermore, total DR5 expression was assayed by western blotting. It was found that PL upregulated DR5 expression in DU145 and MDA-MB-231 in a time- and concentration- dependent manner, although TRAIL by itself also elevated DR5 expression in MDA-MB-231 (Fig. 3b).

To better understand the mechanism of PL-induced DR5 expression, DU145 and MDA-MB-231 cells were pretreated with 1 mM antioxidant N-acetyl Cysteine (NAc) for 1 hr followed by treatment with 15 μM PL for 24 hr. As shown in Fig. 3c, NAc suppressed PL-mediated upregulation of DR5 by quenching intracellular ROS. To confirm whether upregulation of DR5 by PL is essential to sensitize tumor cells to TRAIL, DR5 expression was knocked down via shRNA in DU145 cells. It was found that silencing of DR5 significantly reduced the enhancing effect of PL on TRAIL-induced cell death (Fig. 3d). Therefore, PL potentiates TRAIL-induced apoptosis signaling via ROS-mediated DR5 upregulation at the translational level.

### PL-induced upregulation of DR5 is mediated through MAPK activation

**Figure 4:**
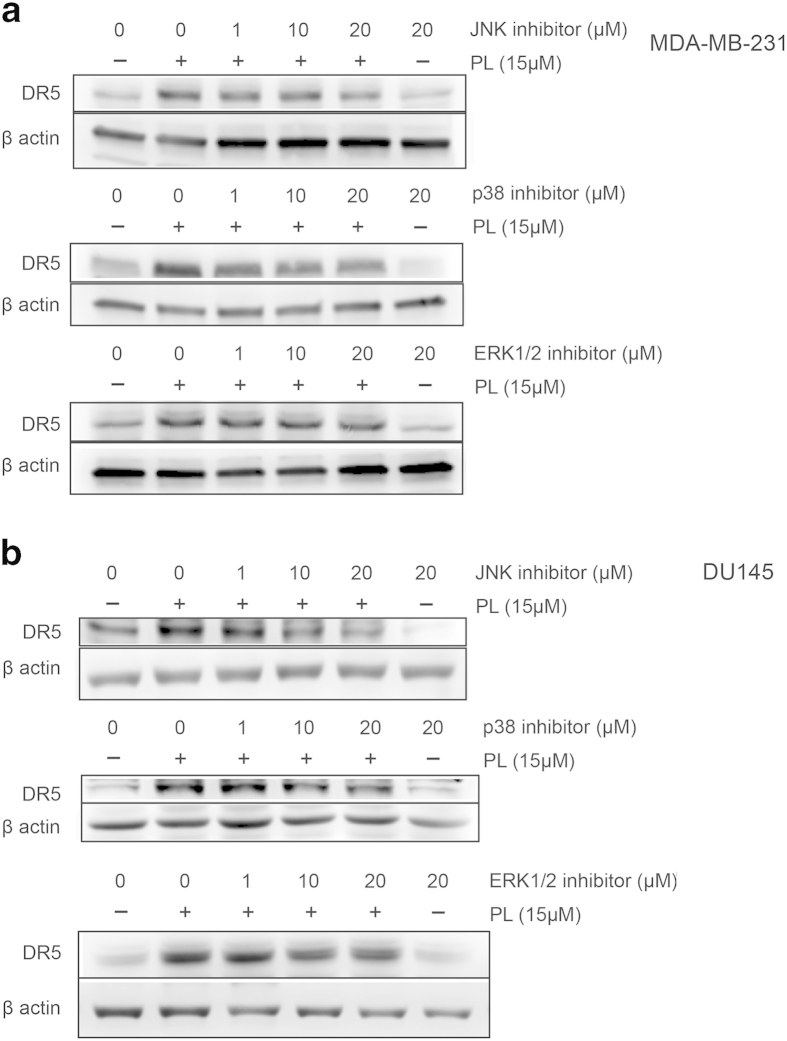
Piperlongumine-induced upregulation of DR5 is mediated through MAPK activation. MDA-MB-231 **(a)** and DU145 **(b)** were pretreated with the indicated concentration of JNK, p38 or ERK inhibitor for 12 hr followed by 15 μM PL for 24 hr. Expression of total DR5 was examined by western blotting. Cropped blots are shown.

Mitogen-activated protein kinases (MAPKs) have been reported to act as sensors for ROS generated in various intracellular stress conditions^25^,^26^. Thus ROS generated from PL treatment likely elevates DR5 expression via MAPKs. To examine whether MAPKs are responsible for PL-induced upregulation of DR5, specific inhibitors for JNK, p38 and ERK1/2 MAPKs were utilized. Pretreatment of MDA-MB-231 or DU145 cells with JNK inhibitor SP600125 suppressed PL-induced upregulation of DR5 in a dose-dependent manner. Similarly, when cells were pretreated with p38 inhibitor SB202190, a dose-dependent decrease in PL-induced DR5 upregulation was observed. In contrast, ERK1/2 inhibitor PD98059 had no such inhibitory effect (Fig. 4a,b). Similar results were also confirmed in HT29 and HCT116 cell lines (Figure S6). Thus, JNK and p38 protein kinases concomitantly upregulate DR5 in cancer cells treated with PL.

### Evaluation of combination therapy in triple-negative breast cancer xenograft model

**Figure 5:**
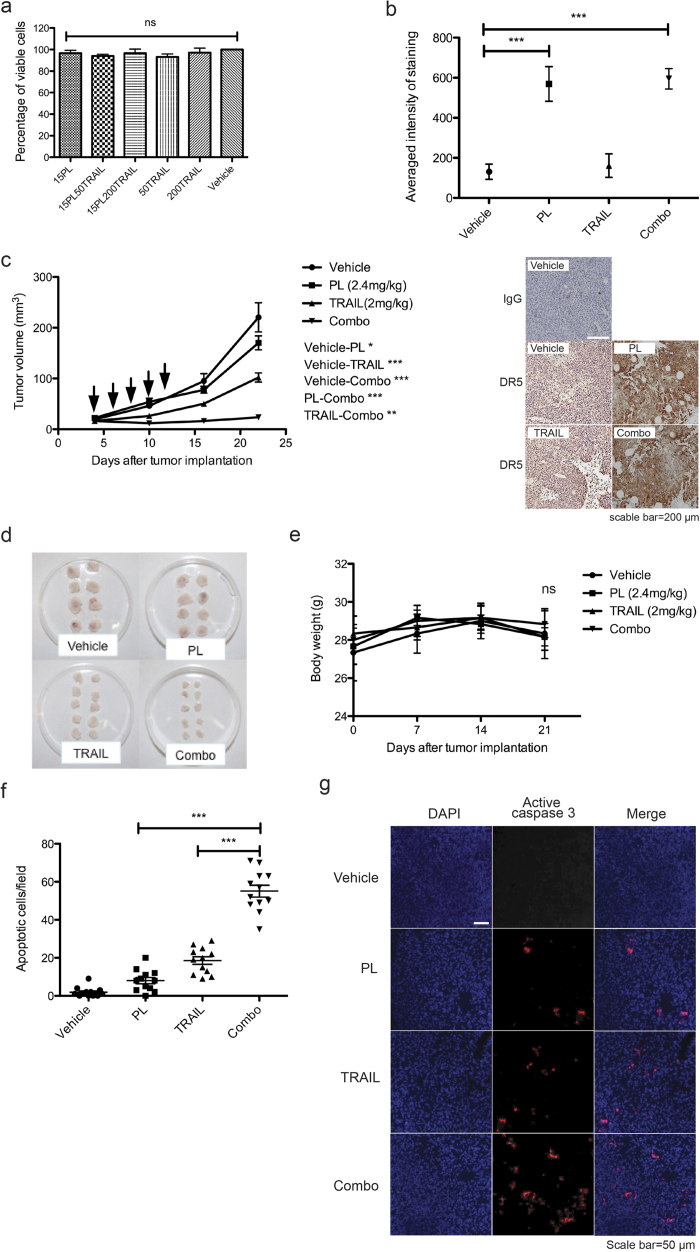
Evaluation of combination therapy in triple-negative breast cancer xenograft model. **(a)** Examination of cytotoxicity of combination therapy in normal cells. Human mammary epithelial cells were treated with the indicated concentration of PL, TRAIL, or their combination *in vitro*. Cell proliferation was measured by MTT assay and compared to vehicle control (DMSO). **(b)** Elevation of DR5 expression in tumors receiving PL. Two doses of vehicle control (DMSO), PL (2.4 mg/kg), TRAIL (2 mg/kg), or combined PL and TRAIL (combo) were intratumorally injected on day 0 and day 1 (n = 3). On day 2, tumors were collected for IHC staining of DR5. The averaged staining intensity was calculated by positive pixel count algorithm and representative IHC images are shown. Rabbit IgG was used as a staining negative control. **(c)** Measurement of subcutaneous tumor growth over three weeks of therapeutic treatment. When tumor size reached 3-5mm in diameter in NSG mice subcutaneously xenografted with MDA-MB-231, vehicle control (DMSO), PL (2.4 mg/kg), TRAIL (2 mg/kg), or combined PL and TRAIL (combo) were administered every other day for a total of five injections. The downward arrows indicate the day of injections. Tumor mass was measured weekly and calculated as described in Materials and Methods. Results are presented as the mean ± SEM for each group (n = 8 or 10). **(d)** Tumors removed at the end of therapy were imaged. **(e)** Measurement of body weight during the therapy. **(f)** Quantification of the number of apoptotic cells in tumor sections. Tissue sections from four tumors in each treatment group were immunostained for active caspase 3. The number of apoptotic cells per fluorescent image was determined for cells identified as positive for active caspase 3 (n = 12). **(g)** Representative fluorescent images are shown. Nuclei were stained with DAPI. All results are mean ± SEM. *, p < 0.05; **, p < 0.01; ***, p < 0.001. ns, no significant difference.

Triple-negative breast cancer (TNBC) is defined as the absence of staining for estrogen receptors, progesterone receptors, and HER2/neu^27^. TNBC is insensitive to some of the most effective therapies available for breast cancer treatment including HER2-directed therapy such as trastuzumab and endocrine therapies such as tamoxifen or the aromatase inhibitors^28^. It was first tested whether the combination therapy induced cytotoxicity of normal cells. No growth inhibition was observed for PL, TRAIL, or their combination when normal breast epithelial cells were tested *in vitro* (Fig. 5a). To confirm that PL can induce upregulation of DR5 *in vivo*, vehicle control, PL, TRAIL or combined PL and TRAIL were injected into subcutaneous tumors (5-8 mm in diameter) of MDA-MB-231 in NOD-SCID gamma (NSG) mice on day 0 and day 1. 24 hr after the second injection, tumors were sectioned and subjected to immunohistochemistry staining for DR5 expression. Whereas DR5 was detected at a basal level in vehicle control and TRAIL treatment groups compared to rabbit IgG isotype-stained slides, it was significantly upregulated in groups receiving PL or combined PL and TRAIL (Fig. 5b). After confirming the safety of combination therapy as well as elevation of DR5 by PL *in vivo*, NOD-SCID gamma (NSG) mice were subcutaneously implanted with human TNBC MDA-MB-231 cells. Four days post implantation when the average diameter of tumors grew to 3-5 mm, NSG mice were intratumorally injected with vehicle control, PL, TRAIL or combined PL and TRAIL (combo) at preclinically relevant dosages every other day for a total of five injections. Weekly measurement of tumor size for three weeks indicated significant inhibition of tumor growth when combined PL and TRAIL were administered (Fig. 5c,d. In the meantime, monitoring of the body weight showed no significant weight loss (Fig. 5e). At the end of therapy, apoptotic cells were quantified in tumor sections corresponding to each treatment group via immunofluorescence staining of active caspase 3. It was found that combined PL and TRAIL resulted in the highest number of apoptotic cells (Fig. 5f,g). Thus, PL and TRAIL combination inhibited tumor growth via potent induction of cancer apoptosis similar to *in vitro* observation.

## Discussion

**Figure 6:**
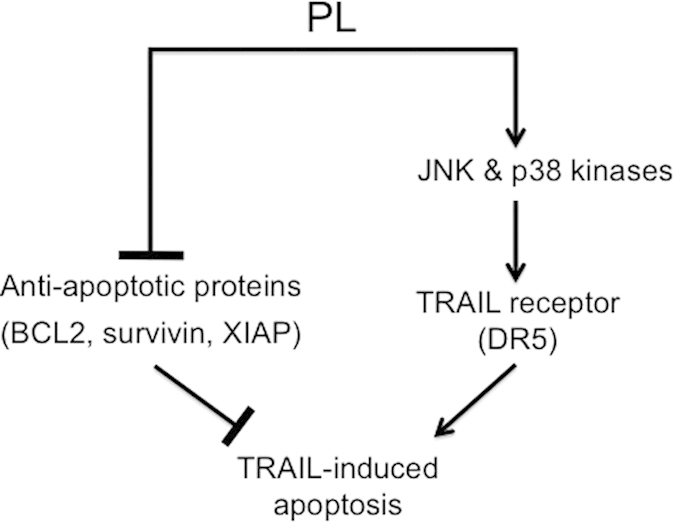
Proposed mechanism of enhanced apoptosis induction by combined PL and TRAIL. PL downregulates anti-apoptotic proteins and increases DR5 TRAIL receptor expression via ROS-mediated activation of MAPKs.

In this study, we provide evidence that PL and TRAIL can synergistically induce apoptosis in cancer cells of various origins. Interestingly, PL was found to enhance the expression of total and membrane-bound DR5, a main TRAIL receptor for TRAIL-induced apoptosis signaling. The upregulation of DR5 was abolished when cancer cells were pretreated with the anti-oxidant N-acetyl cysteine. Furthermore, it was shown that the enhanced DR5 expression caused by PL is dependent on the activation of mitogen-activated protein kinases, JNK1/2 and p38. Thus, we propose two different mechanisms to explain the synergistic effect of combined PL and TRAIL in cancer cells: downregulation of anti-apoptotic proteins, and upregulation of TRAIL receptor DR5 via ROS-mediated activation of MAPKs (Fig. 6).

Although most anti-tumor agents are classified according to their putative mechanism of action, in reality the mechanism of action may be multi-factorial. For example, doxorubicin has been known to generate oxidant stress^29^. Based on our results, we found that both PL and Dox are dependent on ROS to synergize with TRAIL for enhanced apoptosis induction. Additionally, ROS drives the toxicity of PL by itself while Dox does not rely on ROS for its own toxicity. However, such observation should not limit the potential clinical application of PL in combination with TRAIL, although Dox presented similar efficacy when combined with TRAIL. For example, it has been shown that cancer patients experience differential responses to the side effects of Dox in clinical treatments^30^,^31^. Although PL has not been evaluated clinically, its safety in preclinical studies including previous publications and our current work suggests PL and TRAIL combination as a viable option when other TRAIL combination therapies are not effective or are found to cause side effects in certain cancer patients.

TNBC represents one of the most challenging breast cancer subtypes for effective targeted therapy due to the absence of common receptors expressed on other breast cancer subtypes. Whereas resistance to cell cycle inhibition drugs such as doxorubicin and paclitaxel have been detected in TNBC^32^, the current study tested the combination of PL and TRAIL in a xenograft mouse model using the TNBC cell line MDA-MB-231. Consistent with the synergistic effect observed *in vitro*, this combination therapy significantly inhibited the growth of MDA-MB-231 even one week after discontinuing the treatment. The presented *in vivo* trials involved intratumoral (i.t.) injection of the therapeutics since this technique has been extensively evaluated over the past few decades^33^,^34^,^35^,^36^,^37^. An advantage of using this method is that i.t. injection allows for extremely high doses of drug within the tumor with minimal systemic toxicity. Additionally, this approach allows one to directly evaluate the synergistic effect of the PL and TRAIL without the results being affected by the pharmacokinetic factors associated with intravenous injection. We acknowledge, however, that this is also a limitation because the delivery method provides little insight into the therapy’s systemic effectiveness. In previous trials utilizing a systemic delivery approach, not reported here, we have found that PL did not accumulate at sufficient levels at tumors. The pharmacokinetics of the combination therapy will be the subject of future work. Despite this, the observed synergistic therapeutic activity identifies PL and TRAIL as a promising combination therapy for potentially several types of cancer using an i.t. delivery approach.

## Materials and Methods

### Cell lines and Mice

Human colon cancer HT29, prostate cancer DU145, breast cancer MDA-MB-231 and normal primary mammary epithelial cells were obtained from American Type Culture Collection (ATCC) (Rockville, MD, USA). Human colon cancer HCT116 was kindly provided by Dr. Xiling Shen (Cornell University, Ithaca, NY, USA). MDA-MB-231 cells were cultured in DMEM (Invitrogen, Grand Island, NY, USA) with 10% FBS and the other cell lines were cultured in RPMI 1640 (Invitrogen) with 10% FBS. Primary mammary epithelial cells were cultured in Mammary Epithelial Cell medium (ATCC) and were used up to passage number 6. Six to eight-week old female NOD SCID gamma mice were purchased from Jackson Laboratory (Bar Harbor, ME, USA). Mice were housed in a SPF barrier animal facility at Cornell University.

### Chemicals and Antibodies

Piperlongumine and doxorubicin were purchased from Cayman Chemical Company (Ann Arbor, MI, USA). Soluble recombinant human TRAIL for *in vitro* work was purchased from PeproTech (Rocky Hill, NJ, USA). His-tagged TRAIL for *in vivo* work was produced and purified as previously described^38^. JNK, ERK and p38 inhibitors were obtained from LC laboratories (Woburn, MA, USA). The following chemicals or kits were used for assaying cell proliferation and apoptosis: MTT (AMRESCO, Solon, OH, USA), Crystal Violet (Acros Organics, Pittsburgh, PA, USA), and TACS® Annexin V-FITC Kit (Gaithersburg, MD, USA). Antibodies for western blotting or flow cytometry were: mouse anti-caspase 3 (Novus Biologicals, Littleton, CO, USA), mouse anti-β actin (Santa Cruz Biotech, Santa Cruz, CA, USA), PE-conjugated anti-DR4 (Santa Cruz Biotech), PE-conjugated anti-DR5 (R&D Systems, Minneapolis, MN, USA), rabbit anti-DR5 (Abcam, Cambridge, MA, USA), goat anti-rabbit IgG-HRP (Santa Cruz Biotech) and goat anti-mouse IgG-HRP (Santa Cruz Biotech). Human DR5 shRNA was purchased from Sigma (St. Louis, MO, USA).

### Flow cytometry

Cells were detached with enzyme-free Gibco® Cell Dissociation Buffer (Invitrogen) and suspended at a concentration of 5 × 10^5^ cells in 100 μL cold PBS/1% bovine serum albumin (BSA). Primary antibodies or corresponding isotype control antibodies were incubated with cells for 30 min on ice. Following two washes with 1 mL of PBS/1% BSA, fluorescence measurements were collected using an Accuri C6 flow cytometer (BD Biosciences, San Jose, CA, USA). Data were analyzed using the Flow Express software (De Novo Software, Los Angeles, CA, USA).

### Western blotting

Western blotting was performed as previously described^39^. Briefly, whole cell lysates were prepared and separated using 10% SDS-PAGE. Membranes were incubated with primary antibodies and secondary antibodies diluted at 1:1000. Immobilized proteins were detected by using a chemiluminescent HRP substrate (Millipore, Billerica, MA, USA).

### Cell proliferation assay

The effect of PL and TRAIL on cell proliferation was assayed by measuring mitochondrial dehydrogenase activity using MTT as the substrate. After drug treatment, cells were incubated with MTT at a concentration of 0.5 mg/mL, at 37 °C for 3 hr. The purple MTT product was solubilized with DMSO and measured at 570 nm using a BioTek plate reader (Winooski, VT, USA). The effect of PL and TRAIL was also evaluated qualitatively using a crystal violet cell viability assay. After drug treatment, cells were washed with PBS and fixed with methanol. The fixed cells were incubated with crystal violet at a concentration of 0.5% for 20 min at room temperature. The blue crystal violet product was solubilized with a 1% SDS solution.

### Evaluation of synergistic effect by Jin’s formula

The synergistic effect of combined PL and TRAIL was analyzed by Jin’s formula^40^. The formula is Q = E_a+b_/(E_a_ + E_b_ − E_a_ × E_b_), where E_a+b_, E_a_ and E_b_ are the average inhibitory effects of the combination treatment, PL only and TRAIL only, respectively. In this method, Q < 0.85 indicates antagonism, 0.85 < Q < 1.15 indicates additive effects, and Q > 1.15 indicates synergism. The E_a+b_, E_a_ and E_b_ quantities were obtained from MTT assay.

### Measurement of ROS production

Cells were first treated with PL, Dox or DMSO for 6 hr and then loaded with 1 μM of CM-H2DCFDA (Invitrogen, Carlsbad, CA, USA) in PBS for 10 min at 37 °C. Afterwards, cells were allowed to recover in growth medium for 15 min at 37 °C. Cells were analyzed using a flow cytometer. In groups receiving pretreatment of NAc, 1 mM NAc was added 1 hr prior to incubation with PL, Dox or DMSO. Histograms are representative of two separate experiments.

### Lentiviral production and transduction

Lentiviral vectors (scrambled control shRNA and shRNA against DR5) and packaging helper plasmids pMD2G and psPAX2 were transfected into HEK293T cells by TransIT®-LT1 Transfection Reagent (Mirus Biology, Madison, WI, USA). Virus supernatants were harvested at 48 hr and 72 hr after transfection. Virus supernatants were mixed with target cells in the presence of 8 μg/ml polybrene (Santa Cruz Biotech) for 24 hr. Afterwards, fresh media was added and cells were selected with 1 μg/ml puromycin for one week.

### Animal studies

All mice were handled according to the Guide for the Care and Use of Laboratory Animals in compliance with US- and UK-based guidelines. All experimental procedures and protocols were approved by the Institutional Animal Care and Use Committee of Cornell University (Protocol No. 2011-0051). Hairs at the dorsal area of female NOD SCID gamma (NSG) mice were shaved on the day of injection. For examination of DR5 expression induced by PL, 1 million of MDA-MB-231 cells were implanted subcutaneously. When tumors reached 5-8 mm in diameter, vehicle control (DMSO), PL (2.4 mg/kg), TRAIL (2 mg/kg), or a combination treatment were administered intratumorally, twice for two days. 24 hr after the second administration, tumors were collected and subjected to immunohistochemical (IHC) staining for DR5. Rabbit IgG isotype was used as a control for detection of nonspecific IHC staining. For combination therapy, mice were injected subcutaneously with 100 μL sterile PBS containing 1 million MDA-MB-231 cells. When tumors reached 3-5 mm in diameter, mice received the vehicle control (DMSO), PL (2.4 mg/kg), TRAIL (2 mg/kg), or a combination treatment intratumorally at the indicated time points. Tumor dimensions were determined using a caliper, and the tumor volume (mm^3^) was calculated by the formula: volume = length × width^2^ × 0.5. Mice were euthanized 3 weeks after the inoculation of cancer cells.

### Immunohistochemistry and digital analysis

Sections of 4% paraformaldehyde-fixed paraffin-embedded tumor slides were stained with anti-human DR5 rabbit polyclonal antibody or rabbit IgG control. ABC kit (Vector Laboratories, Burlingame, CA, USA) and DAB kit (Vector Laboratories) were used to develop the signals from antibody staining. Slides were counterstained by hematoxylin (Vector laboratories). Glass slides were scanned with Aperio ScanScope scanner (Leica Microsystems Inc., Lincolnshire, IL, USA) and were analyzed with automated image analysis algorithm Aperio Positive Pixel Count (Leica Microsystems Inc.) using the default set of parameters. Briefly, in each slide, tumor cell areas were distinguished from neighboring stromal cells by the higher nucleus to cytoplasm ratio (N/C ratio) of cancer cells. In selected tumor areas, averaged intensity of staining was acquired through dividing total intensity by the sum of the numbers of weak positive (N_wp_), positive (N_p_) and strong positive pixels.

### Immunofluorescence staining of frozen tumor sections

Freshly collected tumors were embedded in OCT and snap frozen in liquid nitrogen. Tumors were sectioned to a thickness of 10 μm by cryotome and mounted on glass slides. Tissue sections were fixed by ice cold acetone and stained with rabbit anti active caspase 3 (Cell signaling, Danvers, MA, USA) followed by DyLight™ 649 Donkey anti-rabbit IgG (Biolegend, San Diego, CA, USA). Slides were counterstained by DAPI and were imaged on an Olympus fluorescence microscope with Metamorph acquisition software.

### Statistical analysis

All statistical analyses were performed using GraphPad Prism 5.0a for Mac OS X (San Diego, CA, USA). A one-way ANOVA followed by Tukey post test was used to compare statistical significance in the characterization of *in vitro* cell proliferation and tumor mass in the xenograft model.

## Additional Information

**How to cite this article**: Li, J. *et al.* Piperlongumine and immune cytokine TRAIL synergize to promote tumor death. *Sci. Rep.* **5**, 9987; doi: 10.1038/srep09987 (2015).

## Supplementary Material

Supplementary Information

## References

[b1] Mitchison T. J. The proliferation rate paradox in antimitotic chemotherapy. Mol. Biol. Cell 23, 1–6, Doi 10.1091/Mbc.E10-04-0335 (2012). 22210845 PMC3248889

[b2] Galmarini C. M., Mackey J. R. & Dumontet C. Nucleoside analogues: mechanisms of drug resistance and reversal strategies. Leukemia 15, 875–890, Doi 10.1038/Sj.Leu.2402114 (2001). 11417472

[b3] Harbeck N., Ewer M. S., De Laurentiis M., Suter T. M. & Ewer S. M. Cardiovascular complications of conventional and targeted adjuvant breast cancer therapy. Ann. Oncol. 22, 1250–1258, Doi 10.1093/Annonc/Mdq543 (2011). 21112929

[b4] Yeh E. T. H. & Bickford C. L. Cardiovascular Complications of Cancer Therapy Incidence, Pathogenesis, Diagnosis, and Management. J. Am. Coll. Cardiol. 53, 2231–2247, Doi 10.1016/J.Jacc.2009.02.050 (2009). 19520246

[b5] Yeh E. T. H. *et al.* Cardiovascular complications of cancer therapy - Diagnosis, pathogenesis, and management. Circulation 109, 3122–3131, Doi 10.1161/01.Cir.0000133187.74800.B9 (2004). 15226229

[b6] Logue J. S. & Morrison D. K. Complexity in the signaling network: insights from the use of targeted inhibitors in cancer therapy. Gene. *Dev.* 26, 641–650, Doi 10.1101/Gad.186965.112 (2012). 22474259 PMC3323875

[b7] Yingling J. M., Blanchard K. L. & Sawyer J. S. Development of TGF-beta signalling inhibitors for cancer therapy. Nat. Rev. Drug Discov. 3, 1011–1022, Doi 10.1038/Nrd1580 (2004). 15573100

[b8] Koivisto P. *et al.* Androgen receptor gene amplification: A possible molecular mechanism for androgen deprivation therapy failure in prostate cancer. Cancer Res. 57, 314–319 (1997). 9000575

[b9] Spector N., Xia W. L., El-Hariry I., Yarden Y. & Bacus S. Small molecule HER-2 tyrosine kinase inhibitors. Breast Cancer Res. 9, Artn 205Doi 10.1186/Bcr1652 (2007).

[b10] Slamon D. J. *et al.* Human-Breast Cancer - Correlation of Relapse and Survival with Amplification of the Her-2 Neu Oncogene. Science 235, 177–182, Doi 10.1126/Science.3798106 (1987). 3798106

[b11] Trachootham D., Alexandre J. & Huang P. Targeting cancer cells by ROS-mediated mechanisms: a radical therapeutic approach? Nat. Rev. Drug Discov. 8, 579–591, Doi 10.1038/Nrd2803 (2009). 19478820

[b12] Schumacker P. T. Reactive oxygen species in cancer cells: Live by the sword, die by the sword. Cancer Cell 10, 175–176, Doi 10.1016/J.Ccr.2006.08.015 (2006). 16959608

[b13] Raj L. *et al.* Selective killing of cancer cells by a small molecule targeting the stress response to ROS. Nature 475, 231–234, Doi 10.1038/Nature10167 (2011). 21753854 PMC3316487

[b14] Wang Y. *et al.* Piperlongumine induces autophagy by targeting p38 signaling. Cell Death Dis. 4, e824, 10.1038/cddis.2013.358 (2013). 24091667 PMC3824668

[b15] Komarova N. L. & Wodarz D. Drug resistance in cancer: Principles of emergence and prevention. P. Natl. Acad. Sci. USA 102, 9714–9719, Doi 10.1073/Pnas.0501870102 (2005). PMC1172248 15980154

[b16] Tang J. *et al.* Target Inhibition Networks: Predicting Selective Combinations of Druggable Targets to Block Cancer Survival Pathways. Plos Comput. Biol. 9, Artn E1003226Doi 10.1371/Journal.Pcbi.1003226 (2013). PMC3772058 24068907

[b17] Wang H., Li F., Du C., Mahato R. I. & Huang Y. Doxorubicin and Lapatinib Combination Nanomedicine for Treating Resistant Breast Cancer. Mol. Pharm. 10.1021/mp400687w (2014). 24405470

[b18] Bellail A. C., Qi L., Mulligan P., Chhabra V. & Hao C. TRAIL agonists on clinical trials for cancer therapy: the promises and the challenges. Rev. Recent Clin. Trials 4, 34–41 (2009). 19149761 10.2174/157488709787047530

[b19] Wang S. & El-Deiry W. S. TRAIL and apoptosis induction by TNF-family death receptors. Oncogene 22, 8628–8633, 10.1038/sj.onc.1207232 (2003). 14634624

[b20] Wang S. The promise of cancer therapeutics targeting the TNF-related apoptosis-inducing ligand and TRAIL receptor pathway. Oncogene 27, 6207–6215, 10.1038/onc.2008.298 (2008). 18931688

[b21] Mitchell M. J., Wayne E., Rana K., Schaffer C. B. & King M. R. TRAIL-coated leukocytes that kill cancer cells in the circulation. P. Natl. Acad. Sci. USA 111, 930–935, 10.1073/pnas.1316312111 (2014). PMC3903223 24395803

[b22] Dimberg L. Y. *et al.* On the TRAIL to successful cancer therapy?Predicting and counteracting resistance against TRAIL-based therapeutics. Oncogene 32, 1341–1350, Doi 10.1038/Onc.2012.164 (2013). 22580613 PMC4502956

[b23] Choo M. K. *et al.* Blockade of transforming growth factor-beta-activated kinase 1 activity enhances TRAIL-induced apoptosis through activation of a caspase cascade. Mol. Cancer Ther. 5, 2970–2976, Doi 10.1158/1535-7163.Mct-06-0379 (2006). 17172402

[b24] Szegezdi E., Cahill S., Meyer M., O’Dwyer M. & Samali A. TRAIL sensitisation by arsenic trioxide is caspase-8 dependent and involves modulation of death receptor components and Akt. Brit. J. Cancer 94, 398–406, Doi 10.1038/Sj.Bjc.6602954 (2006). 16434995 PMC2361137

[b25] Jia Y. T. *et al.* Activation of p38 MAPK by reactive oxygen species is essential in a rat model of stress-induced gastric mucosal injury. J. Immunol. 179, 7808–7819 (2007). 18025227 10.4049/jimmunol.179.11.7808

[b26] Cowan K. J. & Storey K. B. Mitogen-activated protein kinases: new signaling pathways functioning in cellular responses to environmental stress. J. Exp. Biol. 206, 1107–1115, Doi 10.1242/Jeb.00220 (2003). 12604570

[b27] Criscitiello C., Azim H. A., Schouten P. C., Linn S. C. & Sotiriou C. Understanding the biology of triple-negative breast cancer. Ann. Oncol. 23, 13–18, Doi 10.1093/Annonc/Mds188 (2012). 23012296

[b28] Crown J., O’Shaughnessy J. & Gullo G. Emerging targeted therapies in triple-negative breast cancer. Ann. Oncol. 23, 56–65, Doi 10.1093/Annonc/Mds196 (2012). 23012305

[b29] Wang S. *et al.* Doxorubicin induces apoptosis in normal and tumor cells via distinctly different mechanisms. intermediacy of H(2)O(2)- and p53-dependent pathways. J. Biol. Chem. 279, 25535–25543, 10.1074/jbc.M400944200 (2004). 15054096

[b30] Praga C., Beretta G. & Labianca R. Cardiac toxicity from antitumor therapy. Oncology 37 Suppl 1 51–58 (1980). 7005788 10.1159/000225497

[b31] Chlebowski R. T. Adriamycin (doxorubicin) cardiotoxicity: a review. West J. Med. 131, 364–368 (1979). 394479 PMC1271861

[b32] Cobleigh M. A. Other Options in the Treatment of Advanced Breast Cancer. Semin Oncol. 38, S11–Ss16, Doi 10.1053/J.Seminoncol.2011.04.005 (2011). 21600380

[b33] Gochi A., Orita K., Fuchimoto S., Tanaka N. & Ogawa N. The prognostic advantage of preoperative intratumoral injection of OK-432 for gastric cancer patients. Brit. J. Cancer 84, 443–451, Doi 10.1054/Bjoc.2000.1599 (2001). 11207036 PMC2363772

[b34] Han H. D., Byeon Y., Jeon H. N. & Shin B. C. Enhanced localization of anticancer drug in tumor tissue using polyethylenimine-conjugated cationic liposomes. Nanoscale Res. Lett. 9, Artn 209Doi 10.1186/1556-276x-9-209 (2014). PMC4014089 24855464

[b35] Lammers T. *et al.* Effect of intratumoral injection on the biodistribution and the therapeutic potential of HPMA copolymer-based drug delivery systems. Neoplasia 8, 788–795, Doi 10.1593/Neo.06436 (2006). 17032495 PMC1715923

[b36] Sersa G., Stabuc B., Cemazar M., Miklavcic D. & Rudolf Z. Electrochemotherapy with cisplatin: Clinical experience in malignant melanoma patients. Clin. Cancer Res. 6, 863–867 (2000). 10741708

[b37] Xie H., Goins B., Bao A. D., Wang Z. J. & Phillips W. T. Effect of intratumoral administration on biodistribution of Cu-64-labeled nanoshells. Int. J. Nanomed. 7, 2227–2238, Doi 10.2147/Ijn.S30699 (2012). PMC3356223 22619558

[b38] Chae S. Y. *et al.* Improved Antitumor Activity and Tumor Targeting of NH2-Terminal-Specific PEGylated Tumor Necrosis Factor-Related Apoptosis-Inducing Ligand. Mol. Cancer Ther. 9, 1719–1729, Doi 10.1158/1535-7163.Mct-09-1076 (2010). 20515949 PMC3629964

[b39] Li J. *et al.* Human fucosyltransferase 6 enables prostate cancer metastasis to bone. Brit. J. Cancer 109, 3014–3022, Doi 10.1038/Bjc.2013.690 (2013). 24178760 PMC3859952

[b40] Gao F. *et al.* Ulinastatin Exerts Synergistic Effects with Taxotere and Inhibits Invasion and Metastasis of Breast Cancer by Blocking Angiogenesis and the Epithelial-Mesenchymal Transition. Cancer Biother Radio 28, 218–225, Doi 10.1089/Cbr.2011.1122 (2013). PMC3615695 23477357

