# Supplementary material for: Piperlongumine and immune cytokine TRAIL synergize to promote tumor death
Source: Sci Rep. 2015 May 18;5:9987. doi: 10.1038/srep09987 (PMC4649998; doi:10.1038/srep09987)

Piperlongumine and immune cytokine TRAIL synergize to promote tumor death

Jiahe Li[1], Charles C. Sharkey[1], Michael R. King[1,*]

[1]Department of Biomedical Engineering

Cornell University,

Ithaca, New York, USA, 14853

Correspondence should be addressed to M. R. K.

*Correspondence: Michael R. King, Ph.D., 205 Weill Hall, Ithaca, NY 14853.

Phone: 607-255-9803; E-mail: mike.king@cornell.edu

Supplemental figure 1. Qualitative anti-tumor effect of combined PL and TRAIL via crystal violet assay. Cancer cells were treated with the indicated concentrations of PL (0, 5 or 15 μM) and/or TRAIL (0, 50 or 200 ng/mL) in 12-well plates for 24 hr at 37°C. Cell viability was measured using crystal violet assay. Cell lines tested were: **(a)** HT29 (colon cancer), **(b)** DU145 (prostate cancer), **(c)** HCT116 (colon cancer) and **(d)** MDA-MB-231 (breast cancer). Results are qualitatively determined by comparing the darkness of the solution. Darker solution indicates a higher survival rate of the cancer cells receiving the indicated treatment.

Supplemental figure 2. Analysis of synergistic anti-tumor effect of combined PL and TRAIL in cell lines by Jin's formula. The formula is described in Materials and Methods. The inhibitory effects of single and combined treatments were derived from results of MTT assays. PL and TRAIL exerted a synergistic inhibitory effect in the tested cell lines when 15 μM PL was combined with 50 or 200 ng/mL TRAIL (>1.15, synergism).

Supplemental figure 3. ROS elevation induced by PL or Dox and reversion by NAC. MDA-MB-231 and DU145 cells were treated with 15 μM PL, 15 μM doxorubicin (Dox) or DMSO for 6 hr. Cells were also pretreated with 1 mM NAC for 1 hr, followed by PL or Dox for 6 hr. The ROS levels were quantified through flow cytometry using the redox-sensitive fluorescent probe CM-H2DCFDA.

Supplemental figure 4. Dox-induced ROS sensitizes cancer cells to TRAIL. (a) Cancer cells were treated with the indicated concentrations of Dox (10 or 15 μM) and/or TRAIL (50 or 200 ng/mL) in 48-well plates for 24 hr. Cell viability was measured using MTT assay. (b) Reversion of Dox and TRAIL synergy in apoptosis induction by NAc. Pretreatment of DU145 and MDA-MB-231 with 1mM NAc significantly increased viability of cells subjected to combined Dox and TRAIL. All results are presented as the mean ± SEM, n=3; *, $p<0.05$; ***, $p<0.001$.

Supplemental figure 5. qPCR quantification of DR4 and DR5 mRNAs in DU145 cells. Cells were treated with 15 μM PL over 6, 12 and 24 hr. mRNAs of DR4 and DR5 were normalized to that of β actin. Experiments were repeated twice.

Supplemental figure 6. PL-induced upregulation of DR5 is mediated through MAPK activation. HT29 **(a)** and HCT116 **(b)** were pretreated with the indicated concentration of JNK, p38 or ERK inhibitor for 12 hr followed by 15 μM PL for 24 hr. Expression of total DR5 was examined by western blotting. Cropped blots are shown.

Supplemental figure 7. Full blots of figure 2a. Corresponding bands were indicated next to protein name based on molecular weight (MW) of the protein relative to protein ladder (not shown).

Supplemental figure 8. Full blots of figure 3b and 3c. Corresponding bands were indicated next to protein name based on molecular weight (MW) of the protein relative to protein ladder (not shown).

Supplemental figure 9. Full blots of figure 4. Corresponding bands were indicated next to protein name based on molecular weight (MW) of the protein relative to protein ladder (not shown).

Supplemental figure 1

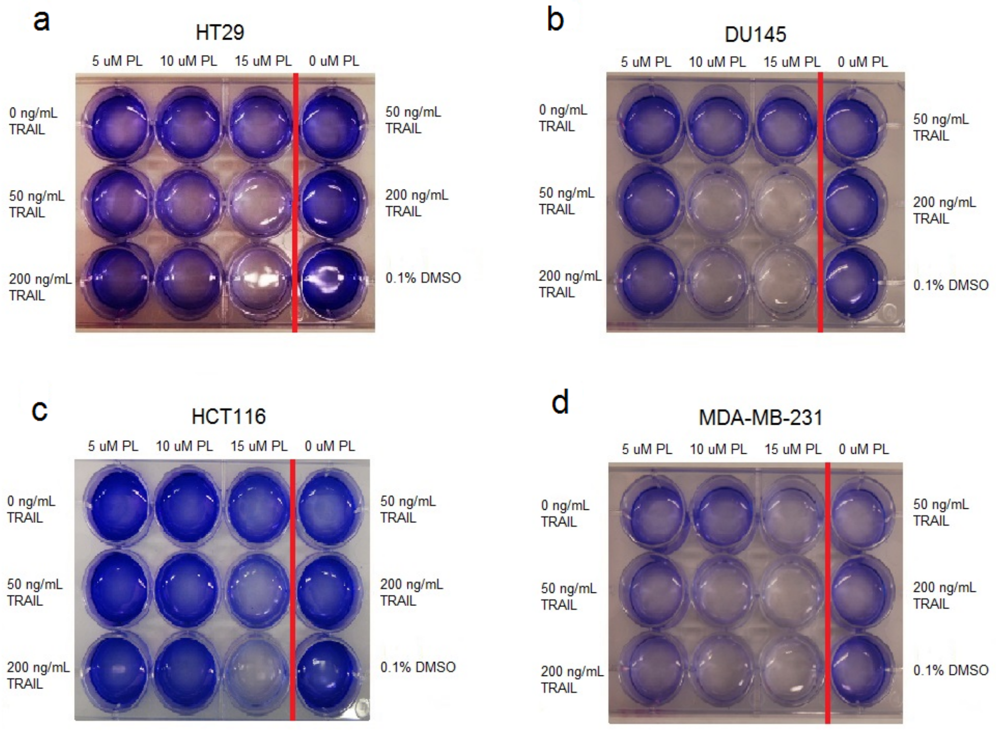

Supplemental figure 2

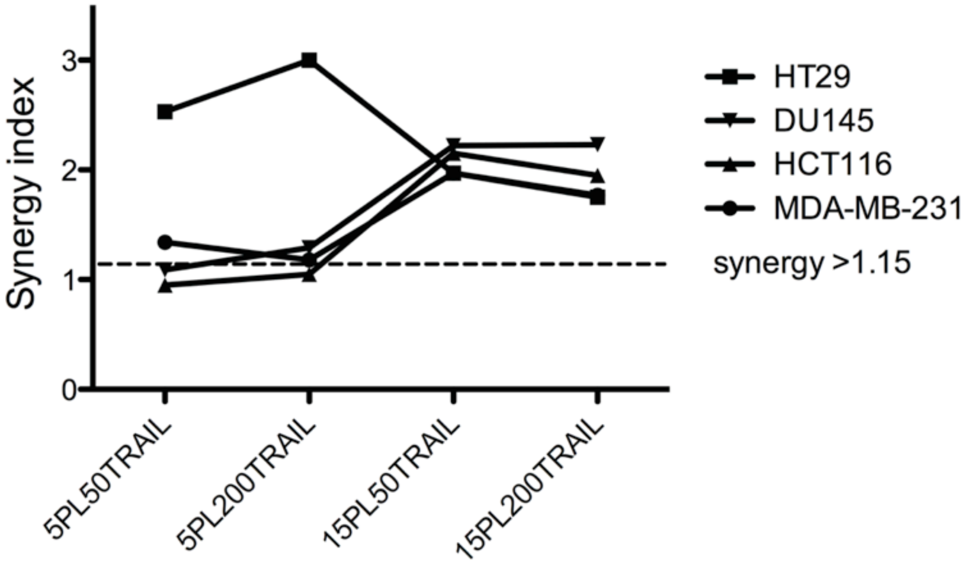

Supplemental figure 3

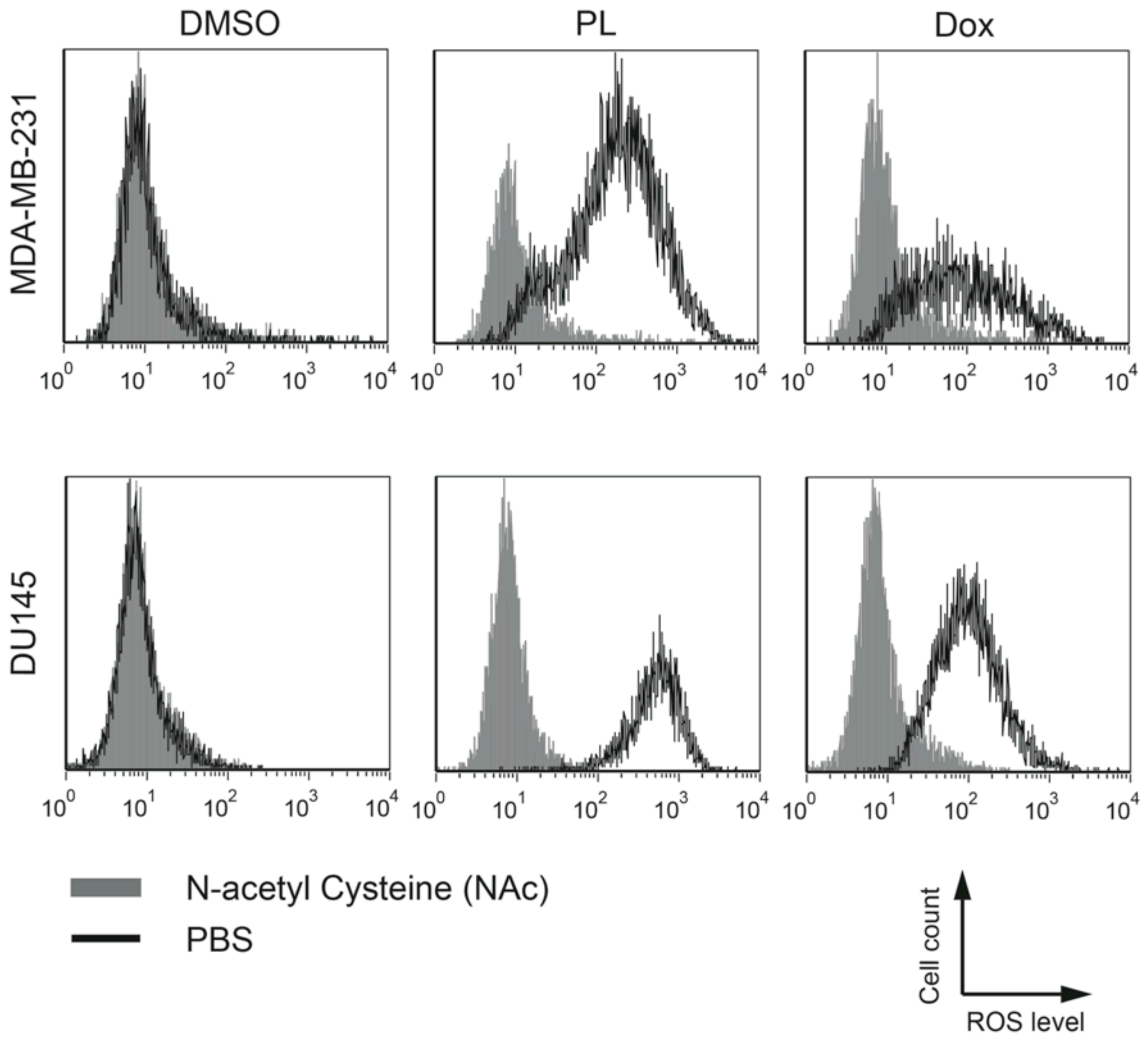

Supplemental figure 4

a

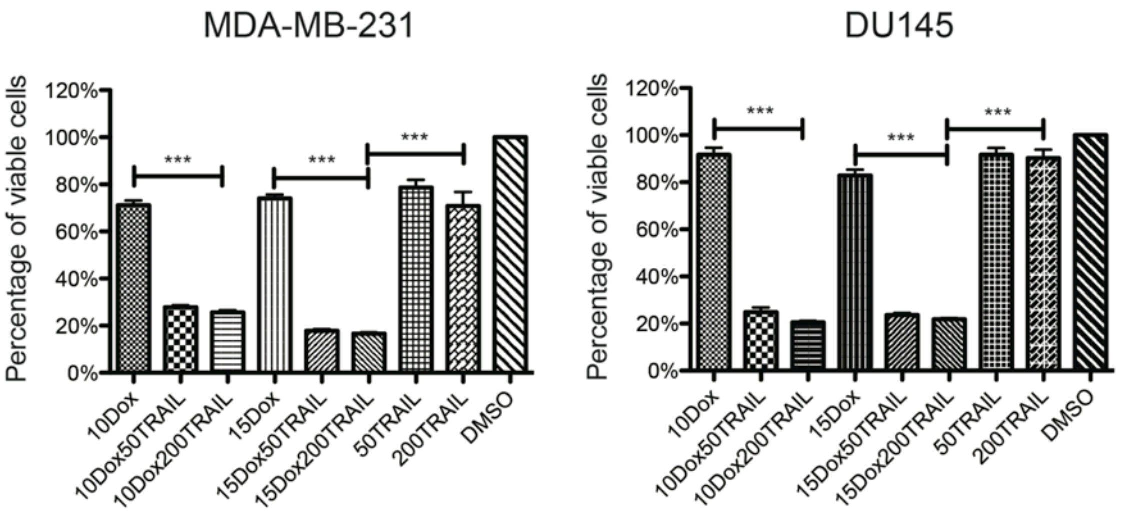

b

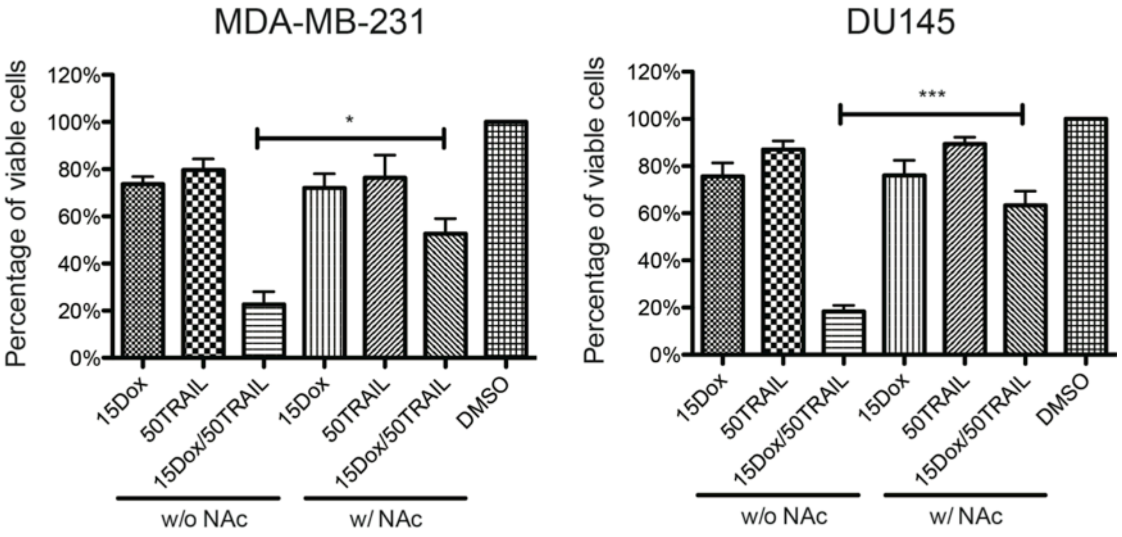

Supplemental figure 5

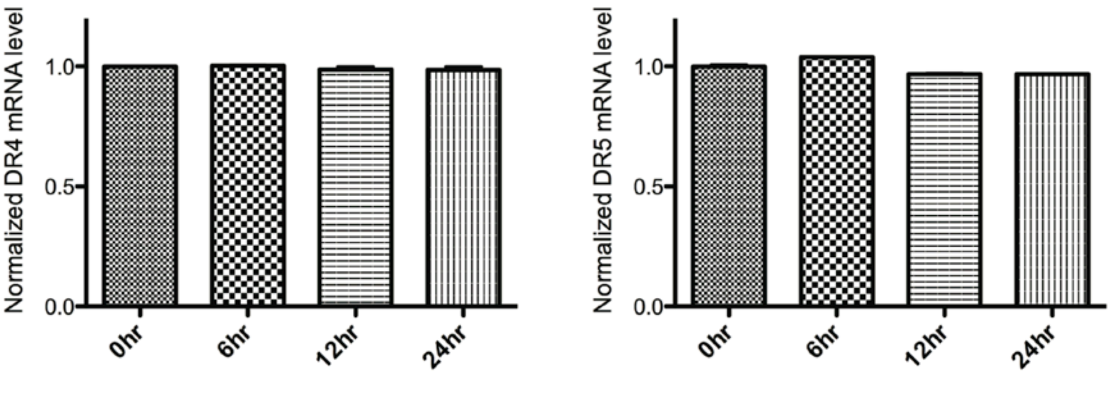

Supplemental figure 6

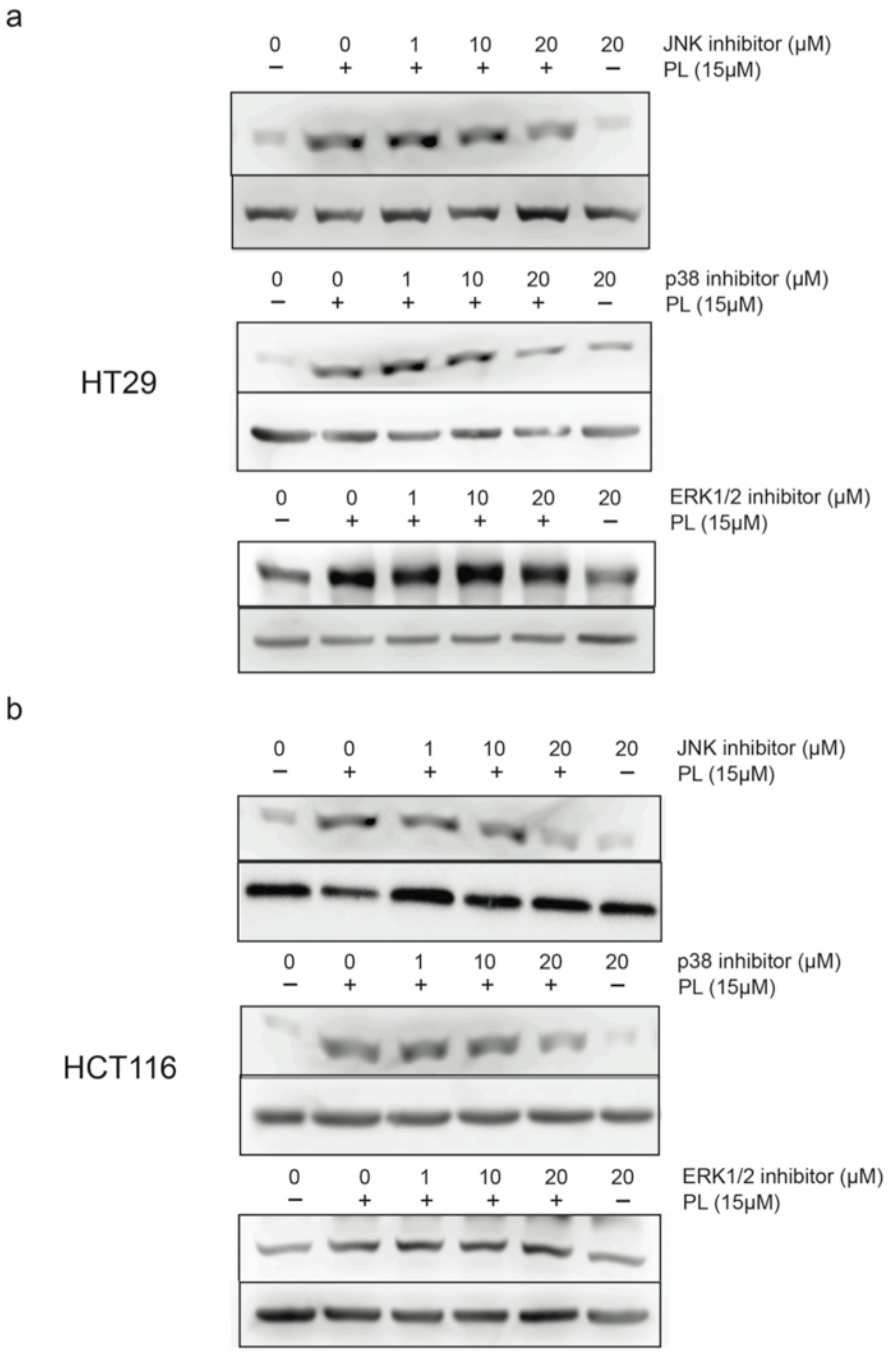

Supplemental figure 7

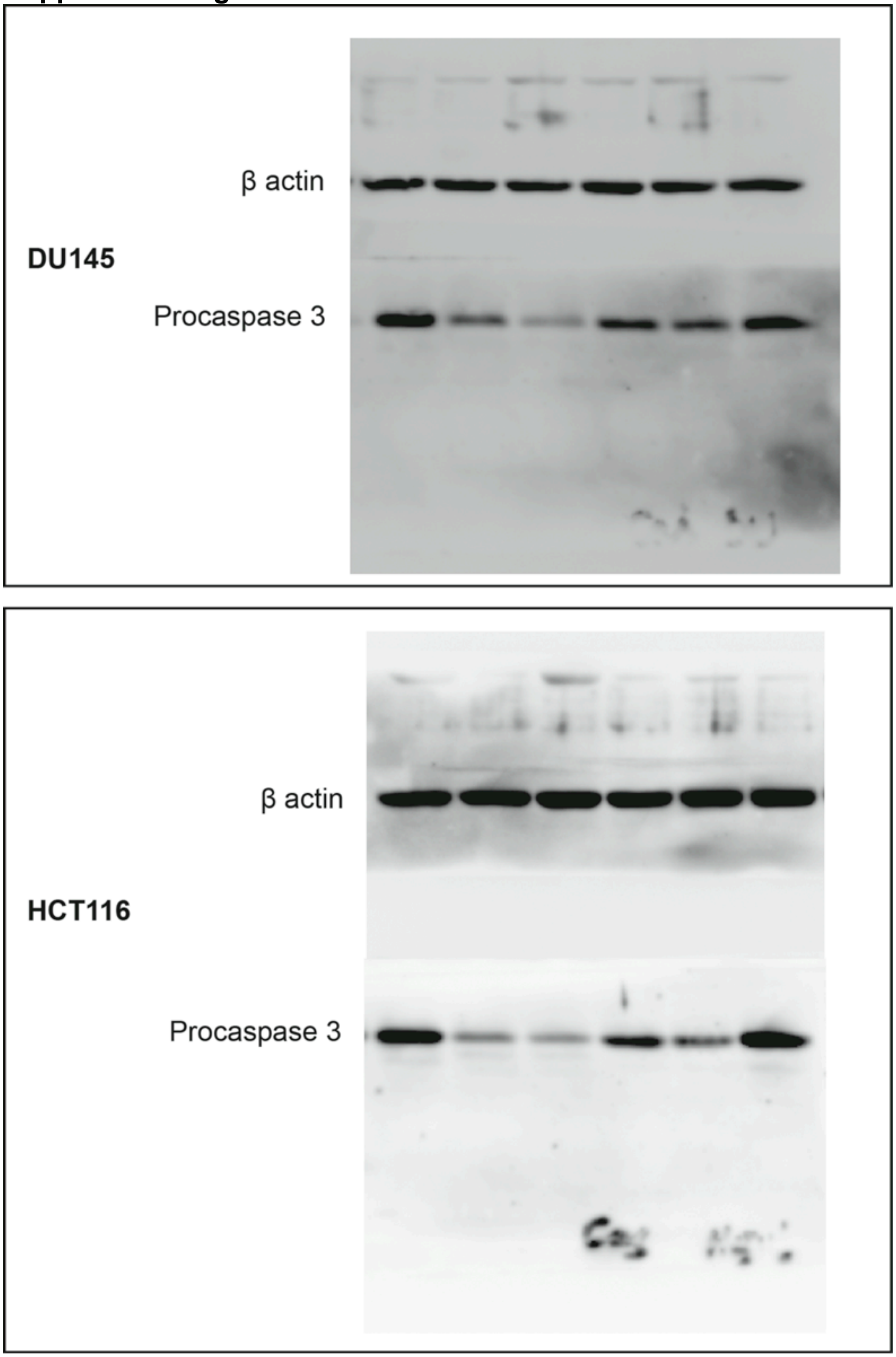

Supplemental figure 8

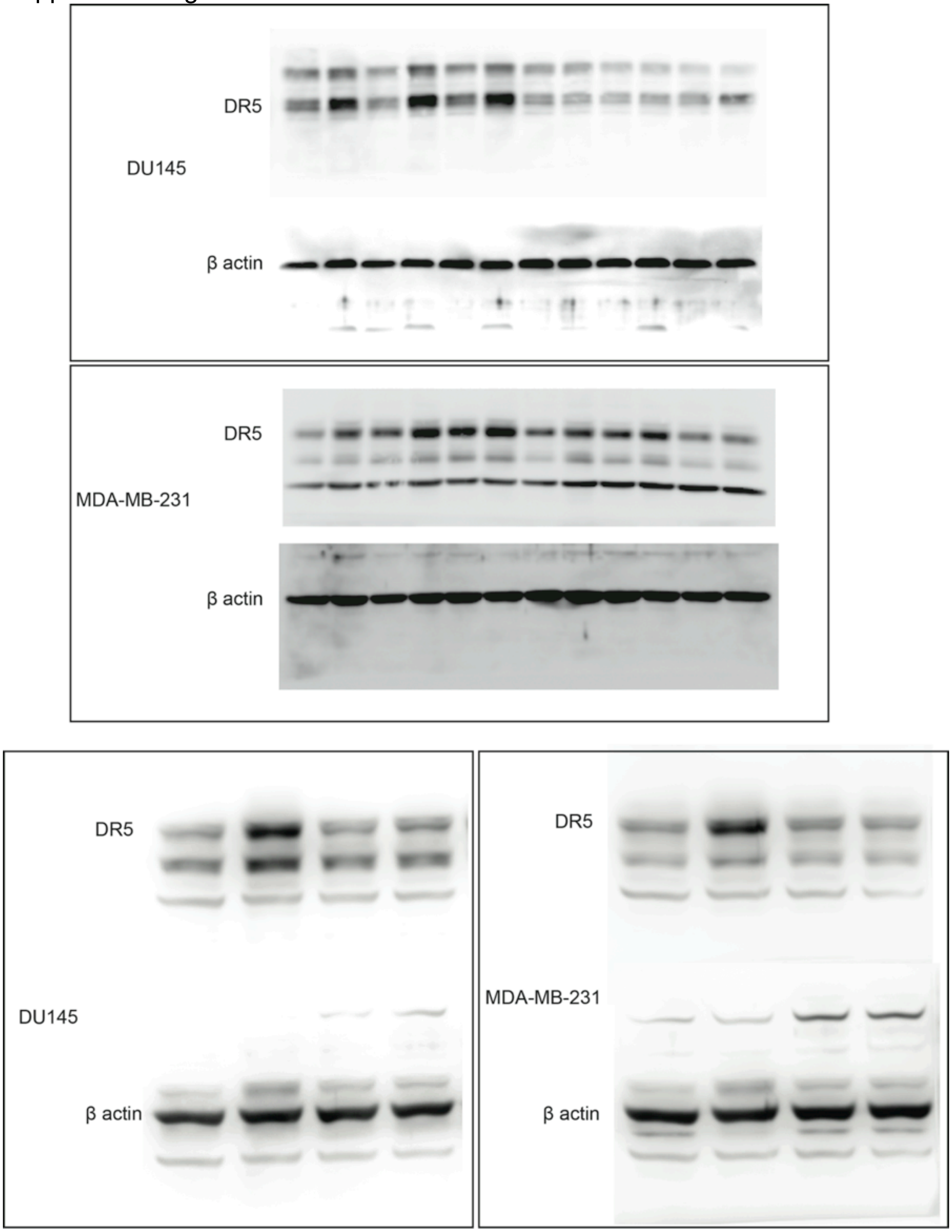

Supplemental figure 9

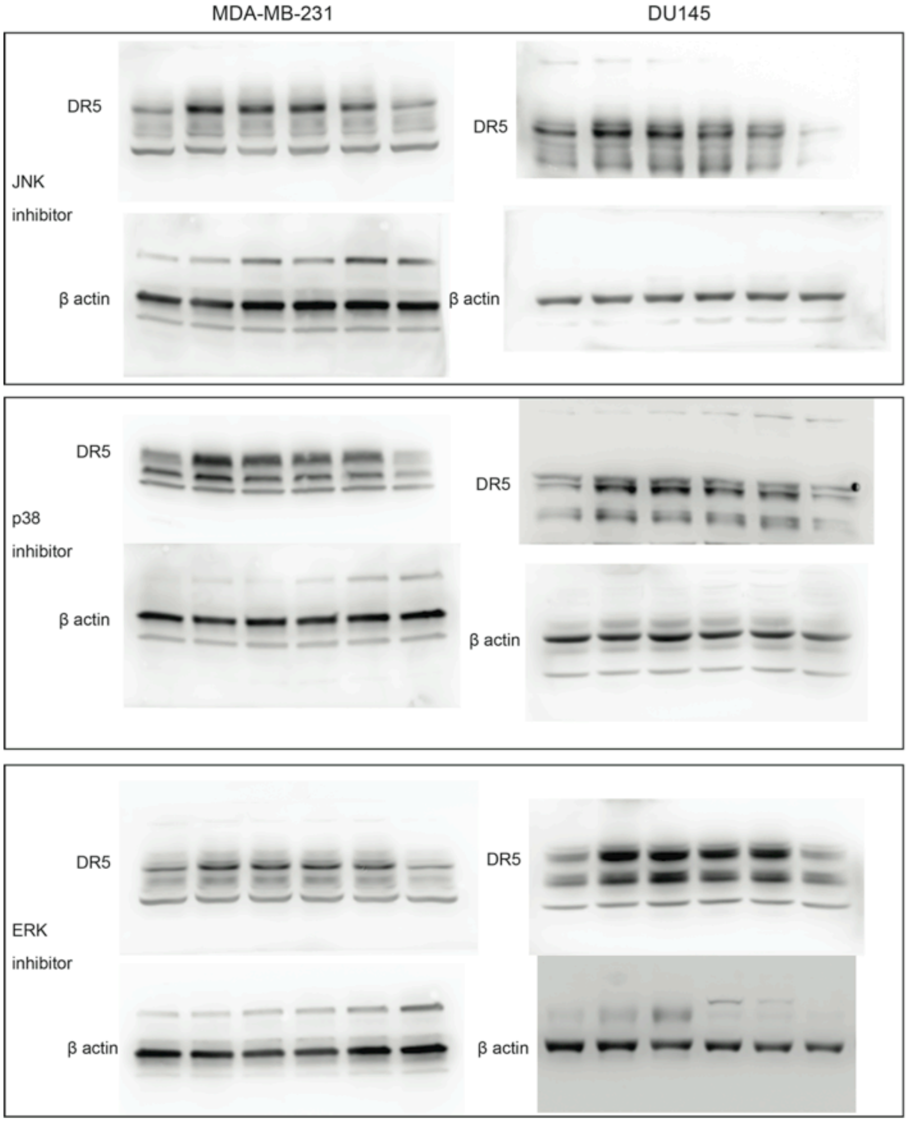

Supplement: Supplementary Information [file srep09987-s1.pdf]
